# Kinetic analysis of [^18^F] altanserin bolus injection in the canine brain using PET imaging

**DOI:** 10.1186/s12917-019-2165-5

**Published:** 2019-11-21

**Authors:** Glenn Pauwelyn, Lise Vlerick, Robrecht Dockx, Jeroen Verhoeven, Andre Dobbeleir, Tim Bosmans, Kathelijne Peremans, Christian Vanhove, Ingeborgh Polis, Filip De Vos

**Affiliations:** 1 Laboratory of Radiopharmacy, Ghent University, Ottergemsesteenweg 460, 9000 Ghent, Belgium; 2 Small animal Departments, Faculty of Veterinary Medicine, Ghent University, Merelbeke, Belgium; 3 Department of Psychiatry and Medical Psychology, Ghent University, Ghent, Belgium; 4 Institute Biomedical Technology – Medisip – Infinity, Ghent University, Ghent, Belgium; 5 Department of Nuclear Medicine, Ghent University Hospital, Ghent, Belgium

**Keywords:** Canine brain, Kinetic modelling, 5HT2a receptor, Mood-disorders

## Abstract

**Background:**

Currently, [^18^F] altanserin is the most frequently used PET-radioligand for serotonin_2A_ (5-HT_2A_) receptor imaging in the human brain but has never been validated in dogs. In vivo imaging of this receptor in the canine brain could improve diagnosis and therapy of several behavioural disorders in dogs. Furthermore, since dogs are considered as a valuable animal model for human psychiatric disorders, the ability to image this receptor in dogs could help to increase our understanding of the pathophysiology of these diseases. Therefore, five healthy laboratory beagles underwent a 90-min dynamic PET scan with arterial blood sampling after [^18^F] altanserin bolus injection. Compartmental modelling using metabolite corrected arterial input functions was compared with reference tissue modelling with the cerebellum as reference region.

**Results:**

The distribution of [^18^F] altanserin in the canine brain corresponded well to the distribution of 5-HT_2A_ receptors in human and rodent studies. The kinetics could be best described by a 2-Tissue compartment (2-TC) model. All reference tissue models were highly correlated with the 2-TC model, indicating compartmental modelling can be replaced by reference tissue models to avoid arterial blood sampling.

**Conclusions:**

This study demonstrates that [^18^F] altanserin PET is a reliable tool to visualize and quantify the 5-HT_2A_ receptor in the canine brain.

## Background

The G-protein coupled 5-HT_2A_ receptor is the main excitatory serotonergic receptor in the brain [1, 2]. Studies in humans and rodents found the highest densities of 5-HT_2A_ receptors in the cortex, mainly in frontal regions. The hippocampus and striatum exhibit lower densities of the receptor, while the cerebellum is virtually devoid of 5-HT_2A_ receptors [1, 3, 4]. The receptor is also widely distributed in peripheral tissues, where it mediates platelet aggregation, capillary permeability and smooth muscle contraction [2, 5].

Besides its influence on numerous physiological functions, the 5-HT_2A_ receptor is believed to be involved in the pathophysiology of several neurological and psychiatric disorders including depression, anxiety disorders, personality disorders, obsessive-compulsive disorder, bipolar disorder, Alzheimer’s disease and schizophrenia [2, 5–13]. Positron emission tomography (PET) is an interesting tool in brain imaging research, permitting the visualization of the 5-HT_2A_ receptor in the living brain by using receptor specific radioligands [14–16]. In vivo study of this receptor is an important field of research as it improves our understanding of the various diseases in which the receptor is implicated. Although several PET radioligands for the 5-HT_2A_ receptor have been evaluated, the clinical use of the majority of these radioligands is limited due to low selectivity or high nonspecific binding [17, 18]. Currently, the selective antagonist [^18^F] altanserin is the most frequently used PET radioligand for 5-HT_2A_ receptor imaging. [^18^F] altanserin is characterised by a high brain uptake, high affinity (k_d_: 0.3 nM) and high selectivity towards the 5-HT_2A_ receptor [19–22]. In human neuroimaging, the in vivo formation of lipophilic radiolabeled metabolites that cross the blood brain barrier complicates kinetic modeling with this radiotracer [15, 17, 20, 21]. However, correction for these radiolabeled metabolites can be achieved by the application of a bolus-infusion protocol with equilibrium imaging [23] or by using a dual-input functional approach [24] but is not feasible in clinical routine. Besides in human clinical PET studies, [^18^F] Altanserin has been used in studies with baboons and rodents. Unlike in humans or baboons, only polar radiometabolites have been identified in rodents [1, 3, 21]. To the authors’ knowledge, PET imaging with [^18^F] Altanserin in dogs has never been demonstrated. However, since they show naturally occurring behavioral disorders which are related and possibly homologues to particular human psychiatric disorders, the dogs might present a valuable animal model for them [25–29]. In addition, dogs have a relatively large frontal cortex in comparison with rodents, which makes imaging studies of this region more feasible [30]. Previously performed SPECT and psychopharmacological studies with dogs have already reported a potential involvement of the 5-HT_2A_ receptor in impulsive aggression, pathological anxiety and other affective behavioral disorders [27, 28, 31, 32]. For example, a decreased binding potential of the 5-HT_2A_ receptor in brain regions assumed to play an important role in the pathophysiology of anxiety disorders was reported in dogs with pathological anxiety [28]. On the contrary, increased binding potential of the 5-HT_2A_ receptor was found in dogs suffering from impulsive aggression [31]. These findings emphasize the role of the 5-HT_2A_ receptor in canine behavioral disorders. Using the advantages of PET imaging over other imaging techniques to study the 5-HT_2A_ receptor in the dogs, its involvement in several behavioral disorders can be further investigated which could help to gain insight in the pathophysiology of certain canine behavioural disorders and would help to guide the treatment of these disorders.

The first objective of this study was to quantify [^18^F] altanserin binding in the canine brain using compartmental modelling and a metabolite corrected arterial input function. A second objective was to investigate whether compartmental modelling can be replaced by reference tissue modelling in future experiments, since this would avoid invasive arterial blood sampling and therefore facilitate application of the radiotracer in future studies.

## Results

### Radiosynthesis

[^18^F] altanserin was obtained after 70 min of synthesis with end of synthesis (EOS) activities of 3.14 ± 1.16 GBq (*n* = 5). High chemical and radiochemical purities of > 99% were achieved with a minimal specific activity of 100 GBq/μmol at EOS.

### Blood input function and metabolites

**Fig. 1:**
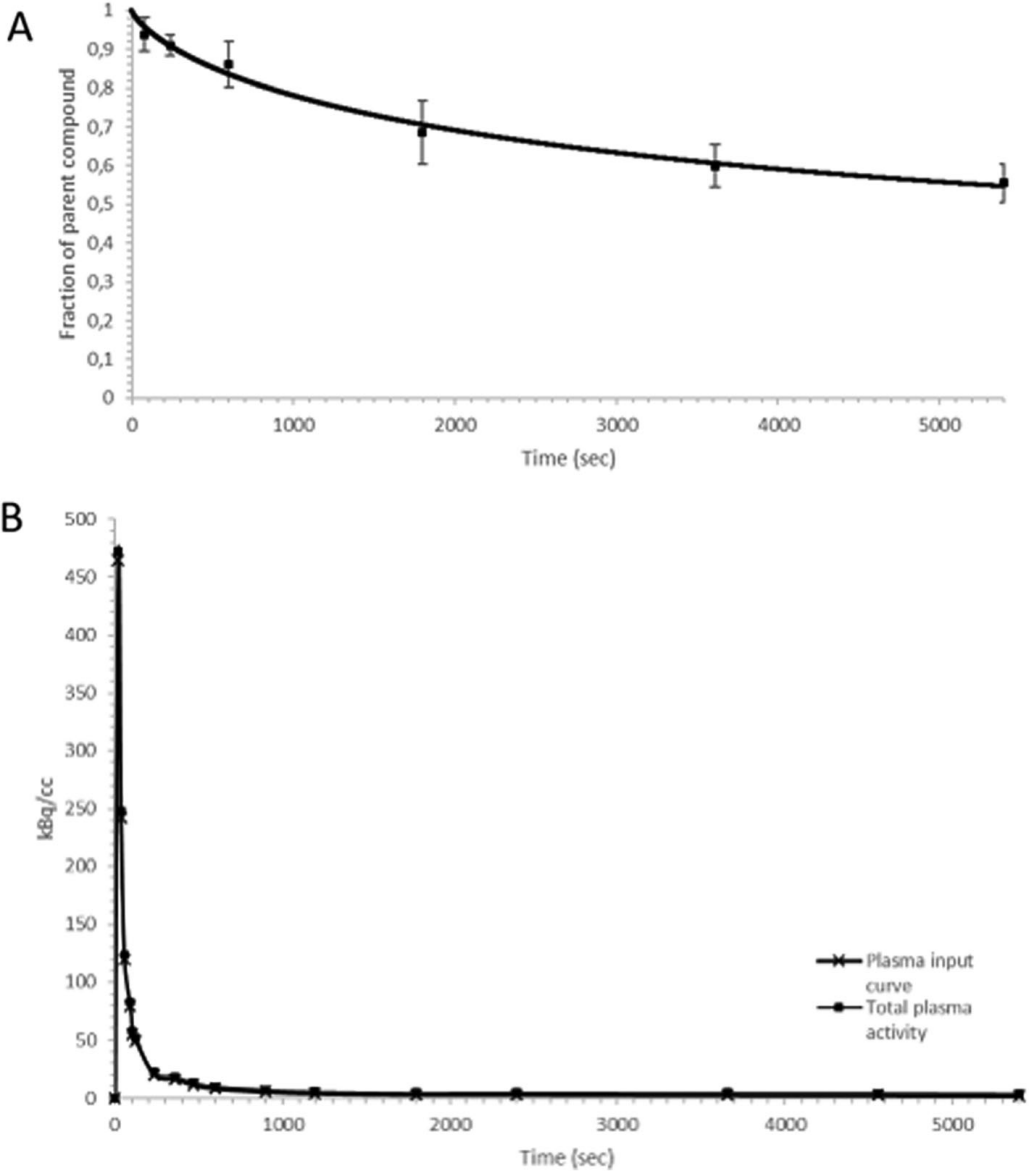
**a**: A Watabe function fitted to the mean fraction of intact [^18^F] altanserin (mean ± SD) in plasma over time for five dogs. **b**: Example of an individual total plasma activity curve and corresponding metabolite corrected plasma input function

The parent compound fraction in plasma over time (mean for five dogs) is visualised in Fig. 1a and illustrates a slow metabolization of [^18^F] altanserin during the PET scan. After 10 min, 86 ± 5.9% of total activity in the blood plasma was intact [^18^F] altanserin and further decreased to 55 ± 4.9% at 90 min. Using HPLC one major unknown polar metabolite (T_R_ = t_0_) could be identified which increased up to 41 ± 3.9% at 90 min. Furthermore, two lipophilic metabolites could be separated from [^18^F] altanserin (T_R_: 10 min) during HPLC analysis: an unknown metabolite (T_R_: 6 min) and a metabolite identified as [^18^F] Altanserinol (T_R_: 8 min). These lipophilic metabolites together never transcended 2% of total radioactivity in plasma. Thereafter, a Watabe function was fitted to the intact fraction of [^18^F] altanserin in the plasma for every individual dog and a metabolite-corrected plasma input curve was calculated using PMOD (Fig. 1b).

### Brain analysis

**Fig. 2:**
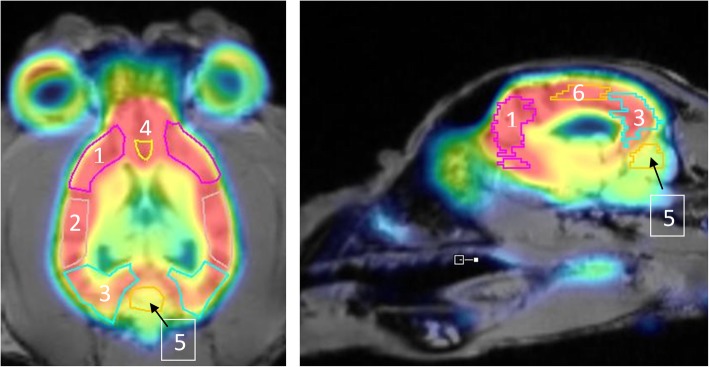
Summed PET image after bolus injection of [^18^F] altanserin co-registered with the MR image. 1: frontal cortex, 2: temporal cortex, 3: occipital cortex, 4: presubgenual cingulate gyrus, 5: cerebellum, 6: parietal cortex. Subgenual cingulate gyrus, ACC and PCC are not included in this figure

**Fig. 3:**
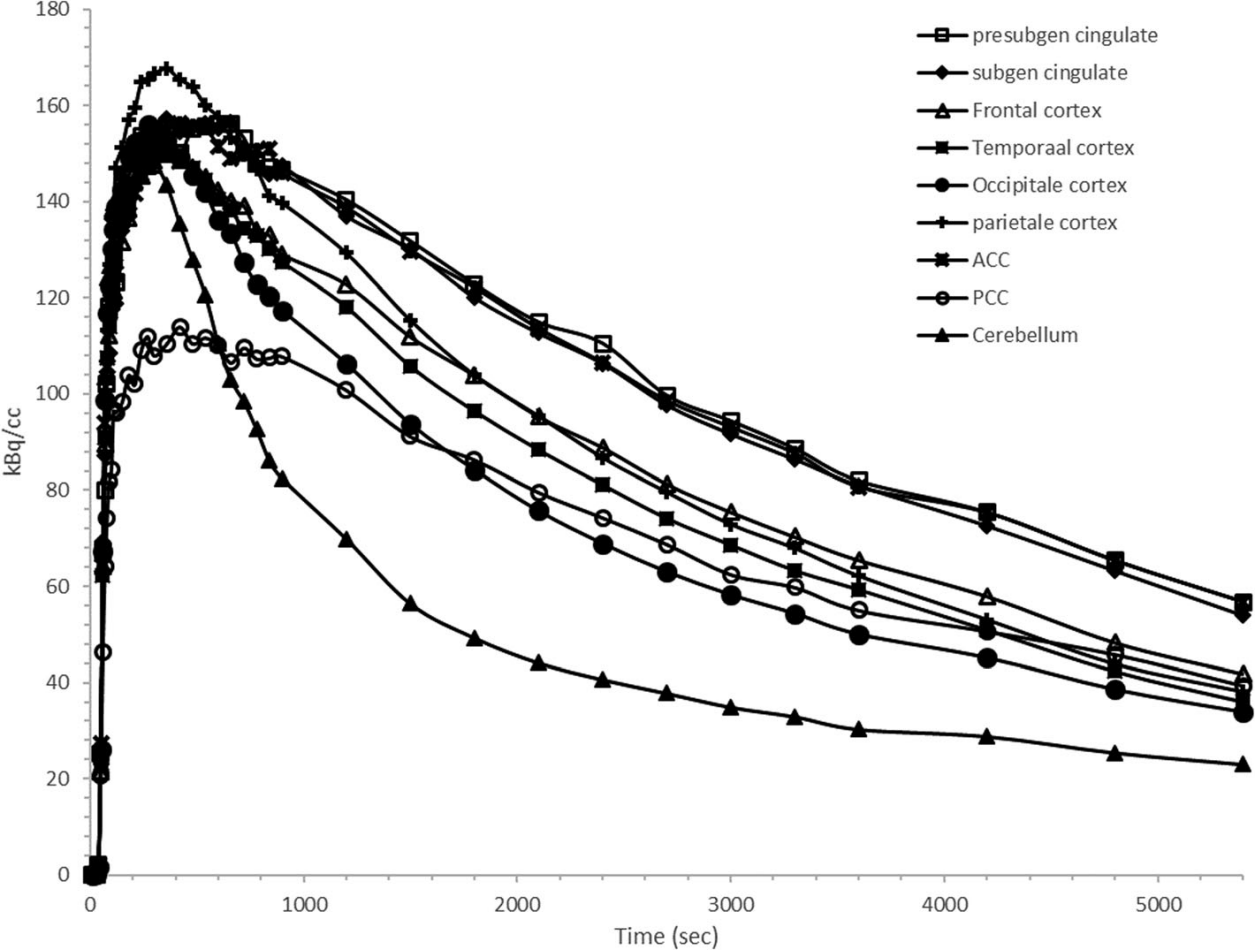
Regional time activity curves after bolus injection of 352 ± 27.7 MBq [^18^F] altanserin for the presubgenual cingulate gyrus, subgenual cingulate gyrus, frontal cortex, temporal cortex occipital cortex, parietale cortex, anterior cingulate cortex (ACC), posterior cingulate cortex (PCC) and cerebellum (i.e. reference region). Data were corrected for radioactive decay

A bolus injection of 352 ± 27.7 MBq [^18^F] altanserin showed a fast uptake in all of the regions followed by a relative fast wash-out. High radioactive uptake was found in the following cortical regions: frontal cortex, parietal cortex, anterior cingulate cortex and subgenual cortex. Moderate uptake was seen in the temporal cortex and occipital cortex. The cerebellum represented the lowest radioactive uptake (i.e. the reference region). Figure 2 represents a summed PET image (frames 1 to 40) co-registered with the MR image. Figure 3 represents the corresponding time-activity curves for the different VOI’s and display a fast equilibrium was achieved after bolus injection of [^18^F]altanserin.

### Kinetic modelling

**Fig. 4:**
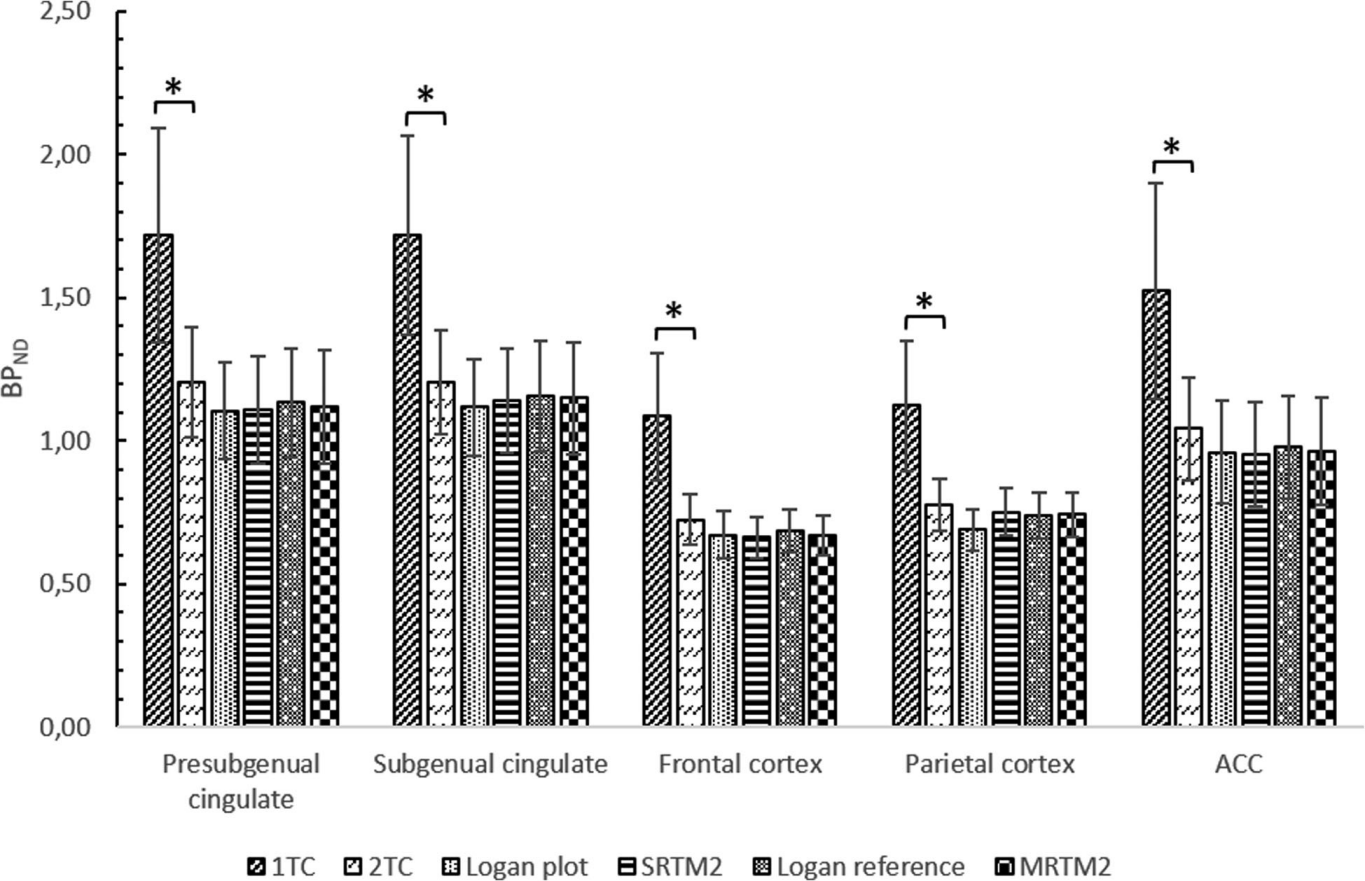
The bars describe the mean value of BP_ND_ ± SD for six different modelling methods in five ROIs (presubgenual cingulate, subgenual cingulate, frontal cortex, parietal cortex and ACC). The modelling methods that are applied are: 1-tissue-compartmental model (1-TC), 2-tissue-comparmental model (2-TC), Logan plot, simplified reference tissue model 2 (SRTM2), Logan reference model and 2 parameter multilinear reference tissue model (MRTM2). The BP_ND_ calculated with the 1-TC model and 2-TC model were significantly different for all ROIs [*p-value < 0.05]. No significant difference was found between the 2-TC model and the Logan plot and all three RTMs [*p*-value > 0.05]

**Fig. 5:**
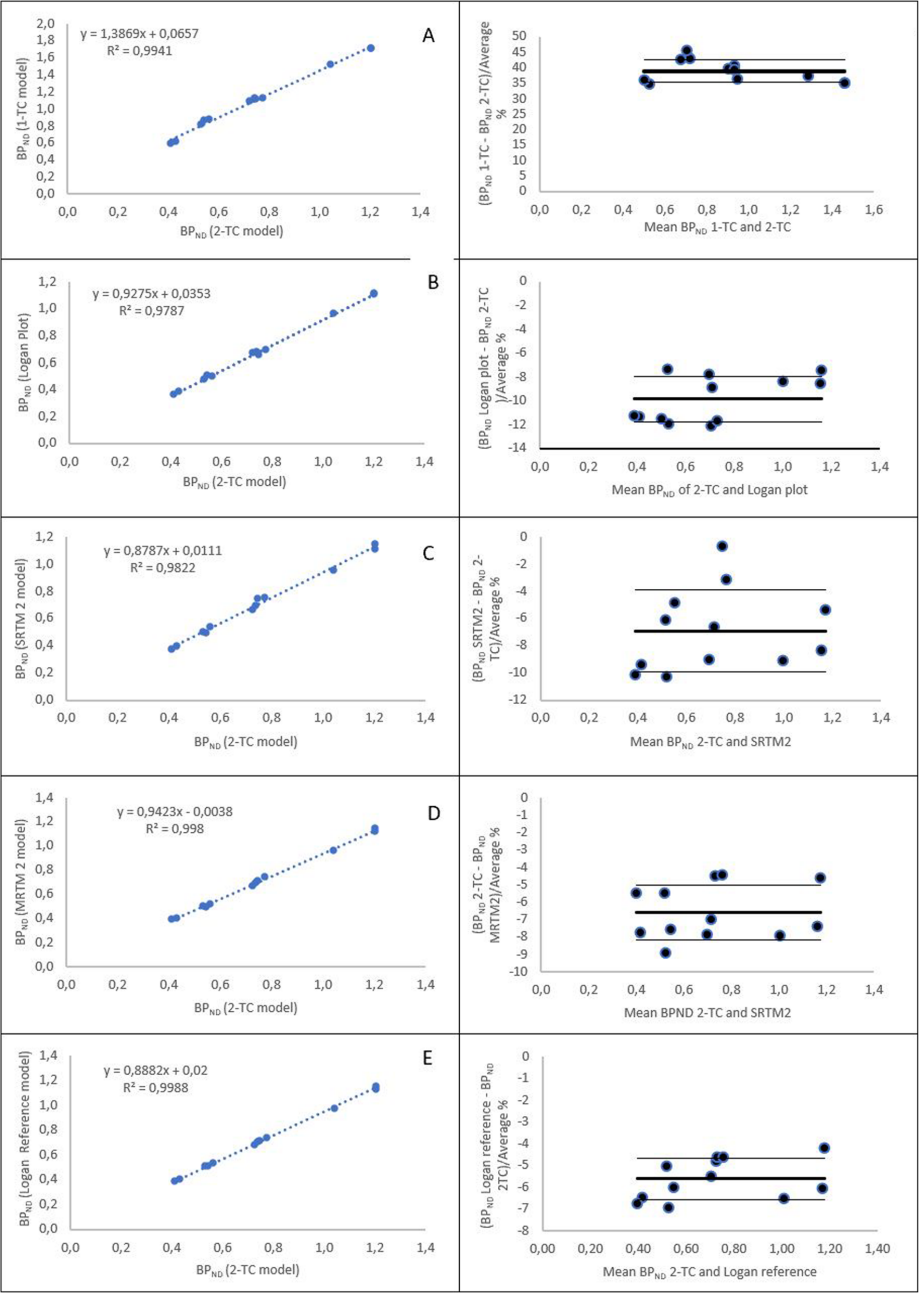
**a**-**e** Graphical comparison of each kinetic model to the 2-TC model presented as a regression analysis (left column) and Bland-Altman plots (right column) where differences are presented as percentage (bold line: mean, narrow line: ± SD). A: 1-TC model vs 2-TC model; B: Logan Plot vs 2-TC model; C: SRTM2 model vs 2-TC model; D: MRTM2 model vs 2-TC model; E: Logan reference model vs 2-TC model

V_T_-, BP_ND_- and AIC-values (mean ± SD) for the six different modelling methods (*n* = 5) are added in Additional data (Additional file 1). Small standard errors (SE) were seen for the 1-TC and 2-TC model with a maximum of 6.56 and 4.47% in the cerebellum, respectively. The 2-TC model showed excellent fits for the target ROI TACs in contrast to the 1-TC model confirmed by the remarkable lower AIC-values observed in all of the brain regions using the 2-TC model compared to the 1-TC model. When plotting the BP_ND_-values of the 1-TC model and 2-TC model in a Bland-Altman plot (Fig. 5) a large overestimation (38.8 ± 3.57%) of the BP_ND_-values was found using the 1-TC model, which is clearly illustrated in Fig. 4. Moreover, a significant difference between de BP_ND_-values of the 1-TC model and 2-TC model was found for all ROIs (left frontal cortex: *p* < 0.001, right frontal cortex: *p* = 0.012, left temporal cortex: p < 0.001, right temporal cortex: *p* = 0.022, left occipital cortex p < 0.001, right occipital cortex *p* = 0.029, left parietal cortex p < 0.001, right parietal cortex *p* = 0.015, anterior cingulate gyrus p < 0.001, posterior cingulate gyrus *p* = 0.004, subgenual cingulate gyrus *p* = 0.038 and presubgenual cingulate gyrus: p < 0.001). Nevertheless, the BP_ND_-values derived from the 1-TC and 2-TC model are well correlated (R^2^ = 0.994) (Fig. 5). Furthermore, a high correlation was also found between BP_ND_-values obtained from the Logan plot compared to the 2-TC model. The Logan plot showed a small BP_ND_ underestimation of − 9.84 ± 1.93% in all regions compared to the 2-TC model but was not significant different (*p*-value > 0.05) (Figs. 4 and 5).

The calculated BP_ND_-values of [^18^F] altanserin in all of the ROIs using the reference tissue models (RTMs) (SRTM2, MRTM2 and Logan reference model) were plotted against those obtained with the 2-TC model (Figs. 4 and 5). The BP_ND_ values of the different reference tissue models were not statistically different from the 2-TC model in any of the ROI’s (*p*-values > 0.05) and showed a SE_max_ of 4.87%. Plotting the obtained BP_ND_ values using Bland-Altman plots a general underestimation of the BP_ND_ could be found for all RTM compared to the 2-TC model (5.63 ± 0.96% (Logan Reference), 6.57 ± 1.57% (MRTM2) and 6.92 ± 3.02% (SRTM2)) (Fig. 5). Nevertheless, all RTMS are highly correlated with the 2-TC model (R^2^(SRTM2) = 0.982, R^2^(Logan Reference) = 0.999, R^2^(MRTM2) = 0.999) (Fig. 5). Additionally, the Bland-Altman plots illustrate constant differences (%) between the BP_ND_ for the RTM and 2-TC model with increasing BP_ND_-value.

### Time stability of the parameters

When artificially shortening the dynamic scanning time to 60 min, BP_ND_ values showed a small reduction of 3.45 ± 2.18% using the 2-TC model and 1.72 ± 3.22% using the Logan Reference model. In contrast, the BP_ND_ values slightly increased when using the SRTM2 model (0.94 ± 2.1%) and MRTM 2 model (1.33 ± 3.33%).

### Semi-quantitative analysis

The results of a semi-quantitative analysis using the contrast of radioactivity (kBq/cc) in each ROI, over the radioactivity (kBq/cc) in the reference region for the different time intervals (10–30 min; 20–40 min; 30–50 min; 40–60 min) are shown in Table 1. When plotting these contrast against the BP_ND_ derived from the 2-TC model, high correlation values were obtained for all of the intervals: R^2^(10–30 min) = 0.955, R^2^(20–40 min) = 0.986, R^2^(30–50 min) = 0.989 and R^2^(40–60 min) = 0.997.

**Table 1:** Activity contrast of ROIs over the cerebellum for 20 min static scans

Region	10–30	20–40	30–50	40–60
Presubgenual cingulate gyrus	0.94 ± 0.18	1.34 ± 0.23	1.46 ± 0.22	1.51 ± 0.22
Subgenual cingulate gyrus	1.01 ± 0.18	1.40 ± 0.22	1.50 ± 0.22	1.54 ± 0.22
Frontal cortex L	0.63 ± 0.10	0.92 ± 0.14	0.99 ± 0.14	1.00 ± 0.15
Frontal cortex R	0.56 ± 0.07	0.83 ± 0.11	0.91 ± 0.12	0.94 ± 0.14
Temporal cortex L	0.52 ± 0.11	0.73 ± 0.12	0.82 ± 0.23	0.77 ± 0.12
Temporal cortex R	0.46 ± 0.06	0.66 ± 0.09	0.70 ± 0.09	0.70 ± 0.10
Occipital cortex L	0.40 ± 0.09	0.55 ± 0.09	0.57 ± 0.08	0.56 ± 0.07
Occipital cortex R	0.37 ± 0.08	0.53 ± 0.07	0.55 ± 0.06	0.54 ± 0.05
Parietal cortex L	0.69 ± 0.16	0.95 ± 0.14	0.98 ± 0.15	0.97 ± 0.15
Parietal cortex R	0.71 ± 0.12	0.95 ± 0.13	0.99 ± 0.13	0.98 ± 0.13
ACC	0.78 ± 0.18	0.16 ± 0.23	1.26 ± 0.23	1.31 ± 0.23
PCC	0.41 ± 0.12	0.65 ± 0.14	0.70 ± 0.13	0.73 ± 0.13
Correlation to 2-TC model	0.971	0.995	0.996	0.997

The contrast ± SD for every ROI over the cerebellum for four different time intervals of 20-min scans (10–30 min; 20–40 min; 30–50 min; 40–60 min). Additionally, the correlation factor (R^2^) with the 2-TC model was presented per time interval

## Discussion

To the authors knowledge, this is the first study that investigates the potential use of [^18^F] altanserin as PET-tracer to quantify the 5HT_2A_ receptor density in dogs. The ability to quantify the 5HT_2A_ receptor in the canine brain is of interest for both canine and human research, since it could improve diagnosis and therapy of behavioral and neuropsychiatric disorders in both species.

Highest radioactive uptake in all brain regions was detected 5 minutes after bolus injection of [^18^F] altanserin and was followed by a relative rapid washout. Time-activity curves were derived from the blood based PET data for all the ROI’s and are represented in Fig. 3. In accordance with studies in humans and rodents, the highest uptake of [^18^F] altanserin was found in the cortical regions. Lowest uptake was found in the cerebellum, supporting the absence of the 5HT_2A_ receptors in the cerebellum and approving its use as reference region in RTMs (Fig. 2). In contrast, a rapid equilibrium was reached after bolus injection of [^18^F] altanserin compared to its very slow kinetics in humans (Fig. 3) [1, 3, 23].

Opposed to imaging studies in humans and baboons, moderate metabolism of [^18^F] altanserin with very low formation of lipophilic blood-brain barrier penetrating metabolites was observed [24, 33]. However, this is in agreement with rodent studies, were only polar metabolites have been reported [3]. After 10 min, [^18^F] altanserin accounted for 86 ± 5.9% of total activity in the blood plasma and further decreased to 55 ± 4.9% at 90 min. One major un-identified polar metabolite (T_R_: t_0_) was found and accounted for 40.8 ± 4.09% of total radioactivity in blood plasma after 90 min scanning. Furthermore, two lipophilic metabolites were detected and could be identified as the known metabolite [^18^F] Altanserinol (T_R_: 8 min). Although previous studies with [^18^F] altanserin in humans and baboons reported up to 20% of certain lipophilic metabolites, in this study the two lipophilic metabolites together never transcended 2% of total radioactivity in plasma. Therefore, there is no need to use a dual-input function compartmental model for BBB permeable metabolites or apply a bolus-infusion protocol [24, 33].

Based on the data obtained with arterial blood sampling, V_T_-, BP_ND_- and AIC-values derived from the 1-TC and 2-TC model were compared with each other. According to the excellent curve fitting and remarkable lower AIC-values in all of the ROIs, the 2-TC model was the compartmental model of choice for [^18^F]altanserin. Although the BP_ND_-values obtained with the Logan plot were highly correlated with those from the 2-TC model (R^2^ = 0.979) and not significantly different from each other, a mean underestimation of 9.84 ± 1.93% compared to the 2-TC model should be kept in mind when using this graphical analysis.

Furthermore, BP_ND_-values were estimated using three different RTMs (SRTM2, MRTM2 and Logan reference model) with the cerebellum as reference region and plotted against the compartmental model of choice, the 2-TC model (Fig. 5). Despite the general underestimation of the reference tissue models compared to the 2-TC model, illustrated by the Bland-Altman plots, (5.63 ± 0.96% (Logan Reference) 6.57 ± 1.57% (MRTM2) and 6.92 ± 3.02% (SRTM2)), all models were highly correlated with the 2TC-model (R^2^(SRTM2) = 0.982, R^2^(Logan Reference) = 0.999, R^2^(MRTM2) = 0.999) (Fig. 5). Moreover, no significant difference could be found between each of the different RTM and the 2-TC model (*p*-values > 0.05). This high correlation between the RTMs and 2-TC indicates that, in future experiments with [^18^F] altanserin in dogs, the invasive blood sampling can be replaced by RTMs, which requires no arterial catheterisation or blood sampling. Hereby, the MRTM2 model or the Logan reference tissue model would be the most appropriate, as they showed the highest correlation (R^2^ = 0.999) with the 2-TC model.

A shorter scanning time is always desirable for the wellbeing of the patient and can significantly increase the throughput of patients/animals per batch of radiotracer. Therefore, the possibility to reduce the scanning time to 60 min was investigated. Shortening the scan time to 60 min instead of 90 min yielded to stable outcomes with a mean bias of less than ±3.5% for both 2-TC model based on arterial input function and RTMs. A shorter scan time could be considered in future experiments, especially when using the SRTM2 or MRTM2 model. Finally, a semi-quantitative analysis using the contrast of radioactivity (kBq/cc) in each ROI, over the radioactivity (kBq/cc) in the reference region showed high correlation values with the 2-TC model for all considered intervals. Highest correlation was found in the interval 40 to 60 min post bolus injection (R^2^ = 0.997). Therefore, a 20 min static PET scan between 40 and 60 min post bolus injection of [^18^F] altanserin could be considered as an alternative method to further simplify the evaluation of 5HT_2A_ receptor binding in future canine studies.

A limitation of this study was mentioned earlier by us and concerns the possible effects of the used anaesthetics on the kinetics of the PET tracer, in this case [^18^F] altanserin [34]. However, the use of anaesthesia cannot be avoided in dogs undergoing PET-scans and a common anaesthesia protocol was used in our study. Since the use of anaesthetics during a PET scan with [^18^F] altanserin in dogs will be inevitable, our results are relevant for future studies. Another limitation of this study is the small number of animals that were included, due to animal welfare restrictions. However, despite the small sample size, highly similar results were found in the individual dogs, therefore, the authors believe that increasing the sample size would probably not provide additional information. Another concern is the fact that only female dogs were included in this study. It is known that pharmacokinetics and metabolism may differ between both sexes, possibly due to differences in sex hormones and other hormones [35, 36]. Therefore, all dogs were neutered before the start of the study. Finally, in order to help calculating the sample size of future PET studies with [^18^F] altanserin, the assessment of the test-retest variability can be performed in a future study.

## Conclusions

In conclusion this first-in-dog study describes a first step in the visualization and quantification of the 5HT_2A_ receptor in the canine brain after bolus injection of [^18^F]altanserin. A moderate metabolism with negligible formation of lipophilic blood-brain barrier penetrating metabolites of [^18^F] altanserin was observed. The study showed that [^18^F] altanserin follows two-tissue compartment kinetics in the canine brain. To avoid invasive blood sampling in future experiments, reference tissue models can be used instead of compartmental modelling, using the cerebellum as reference region. Furthermore, a shorter scan time (60 min) could be considered, especially when using the SRTM2 or MRTM2 model. In order to further simplify the evaluation of the 5HT_2A_ receptor binding, the authors propose to perform a 20-min static PET scan between 40 and 60 min post bolus injection. This study is of great value as the ability to quantify the 5HT_2A_ receptor in the canine brain could help to improve our understanding of the various canine and human diseases in which the receptor is implicated. Better knowledge of the underlying pathophysiology of these diseases can in turn improve diagnosis and treatment strategies.

## Methods

### Experimental animals

The research project was authorized by the Ethical committee of Ghent university (EC approval 17/108) and all manipulations were performed according to good animal practice. Based on previous similar experiments performed in house, five neutered female laboratory beagles (4.7 ± 0.1 years; 11.8 ± 1.19 kg) were included in this project [34, 37]. All dogs were provided by the Faculty of Veterinary Medicine (Ghent University) and were considered to be healthy after general clinical examination. The dogs were fasted for at least 12 h before the start of the experiments but were allowed access to fresh water ad libitum. Following intramuscular (i.m.) premedication with dexmedetomidine (375 μg/m^2^ body surface area, Dexdomitor®, Orion Corporation, Espoo, Finland), the dogs were allowed to relax in a quiet dimmed room. At the PET centre, one of the cephalic veins was catheterized using a 22G over-the-needle catheter and a Lactated Ringer’s solution (Vetivex® 500 mL, Dechra Veterinary products, Heusden-Zolder, Belgium) was infused i.v. at a rate of 5 mL/kg/h. The general anaesthesia protocol was similar to the one used in the study of Pauwelyn et al. 2019 [34]. In brief, anaesthesia was induced with propofol (Propovet®, Abbott laboratories, Queenborough, UK) administered intravenously (i.v.) to effect and maintained with isoflurane (Isoflo®, Abbott laboratories, Queenborough, UK) vaporized in oxygen. Subsequently, the dog was positioned on the bed of the PET/CT scanner and a 22G over-the-needle catheter was placed in one of the dorsalis pedis arteries to enable arterial blood sampling. Heart rate, respiratory rate, end tidal carbon dioxide concentration and arterial haemoglobin oxygen saturation were monitored by pulse oximeter and capnography.

### Radiosynthesis

The radiosynthesis of [^18^F] altanserin was performed on a Synthra RN^+^ module (Synthra GmbH, Hamburg, Germany) with 4 mg nitro-altanserin (ABX, Radeberg, Germany) dissolved in 750 μL anhydrous DMSO as precursor solution (Sigma Aldrich, Germany). Reaction conditions and purification methods were identical as described in Pauwelyn et al. [34] for the PET tracer [^18^F]MPPF.

### Data acquisition

First, a CT scan (120 kV, 35 mA, pitch of 0.7, 20 slices of 3 mm) was acquired, serving as anatomical framework and attenuation correction. Subsequently, each dog received a bolus injection of 352 ± 27.7 MBq (mean: 29.8 MBq/kg) [^18^F] altanserin, and was dynamically scanned with a Siemens Biograph mCT Flow 20 clinical PET/CT imaging system (Siemens, Knoxville, USA) during 90 min. The acquired PET data were reconstructed in 40 frames (12 × 10 s, 6 × 30 s, 10 × 60 s, 9 × 300 s, 3 × 600 s), each consisting of a 512 × 512 matrix with a voxel size of 0.797 × 0.797 × 2 mm, using the TrueX algorithm. All along the PET acquisition, manual arterial blood sampling was performed at 17 time points and collected in and in K_3_EDTA tubes (15, 30 and 45 s, 1, 1.5, 2, 4, 6, 8, 10, 15, 20, 30, 40, 60, 75 and 90 min). Calculating the plasma input curve and % metabolization was similar to previously reported methods [34, 37]. In short, after centrifugation (10 min, 4000 rpm, 4 °C) of the blood samples, radioactivity was measured in the plasma fraction using a gamma counter (Cobra©, Packard, Canberra, Australia) and corrected for decay. The fraction (%) of parent compound in the plasma and the presence of radiolabelled metabolites was measured using an isocratic HPLC method at six different time points (1, 4, 10, 30, 60 and 90 min). Acetonitrile (Sigma Aldrich, Saint Louis, US) (1:2) was added to the remaining plasma and centrifuged (10 min, 4000 rpm at 4 °C). 2 mL supernatant was injected onto a semipreparative HPLC system (Column: Symmetry Prep C18 column (7 μm, 7.8 mm × 300 mm, Waters, Milford, Massachusetts, US); Solvent: 0.05 M NaOAc buffer pH 5/MeOH/THF: 50/32/18 (V/V); flow: 3 mL/min). During 15 min, HPLC eluent was collected into 30 different fractions and was measured for radioactivity in a gamma counter (Cobra©, Packard, Canberra, Australia).

### Image analysis

A 3 T Magnetom Trio Tim system MRI scanner (Siemens, Erlangen, Germany) was used to acquire T1 weighted anatomical images (3D MPRAGE sequence, 176 sagittal slices, TR: 2250 ms, TE: 4.18 ms, TI: 900 ms, parallel acquisition method = GRAPPA acceleration factor: 2, matrix size: 256 × 256, FOV: 220 mm, flip angle: 8 °, voxel size: 1 × 1 × 1 mm^3^). Thirteen regions of interest (ROI) were manually drawn on the MR image using the information of a dog brain atlas [38]: left/right frontal cortex, left/right temporal cortex, left/right occipital cortex, left/right parietal cortex, anterior cingulate gyrus, posterior cingulate gyrus, subgenual cingulate gyrus, presubgenual cingulate gyrus and cerebellum. Afterwards, the MR images were co-registered with the acquired PET/CT images. As described in Pauwelyn et al. [34], for each of the ROIs, time-activity curves were retrieved, a Watabe function was fitted to the % metabolization curve for each dog individually and added to the corresponding plasma input function resulting in a metabolite-corrected plasma input function [39].

### Kinetic modelling

The total volume of distribution (V_T_), was calculated for the different ROIs using the one- and two-tissue compartment (1-TC and 2-TC) model [40] and the Logan plot [41] using PMOD software. The following equations describe how the V_T_ can be calculated for 1-TC and 2-TC model from the obtained rate constants [42].

One-tissue compartment model:

1 $$ \mathrm{VT}=\frac{K1\ }{k2} $$

Two-tissue compartment model:

2 $$ \mathrm{VT}=\frac{K1}{k2}\left(1+\frac{k3}{k4}\right)\kern0.5em $$

Consequently, for both compartmental models and the Logan plot, the non-displaceable binding potential (BP_ND_), referring to the ratio at equilibrium of specifically bound radioligand to that of non-displaceable (ND) radioligand in tissue [42], was calculated. In order to determine the fraction of non-displaceable binding, a region devoid of the targeted receptors (i.e. a reference region) needs to be defined. The cerebellum has been used as reference region in different clinical studies with [^18^F] altanserin [3, 14, 23]. The BP_ND_ for the compartment models and Logan plot can be calculated as follows [42]:

3 $$ \mathrm{BPND}=\frac{V\mathrm{T}}{V\mathrm{ND}}-1 $$

V_ND_ representing the volume of distribution of the non-displaceable radioligand.

Using the Akaike Information Criterion (AIC) value, the goodness-of-fit for each compartment model was evaluated [43]. In order to correct the brain activity for the contribution of plasma activity, the cerebral blood volume in the regions of interest was assumed to be 0.05 mL/cm^3^.

Three RTMs were considered: the 2-step simplified reference tissue model (SRTM2) [44], the 2 parameter multilinear reference tissue model (MRTM2) [45] and the Logan reference tissue model [46]. The capability to reproduce the previous calculated BP_ND_ obtained with the Logan plot and the compartmental models with the RTMs were analysed. The BP_ND_-values were calculated using a fixed k_2_’-value based on the mean k_2_’-value of seven high binding regions: presubgenual cingulate gyrus, subgenual cingulate gyrus, left/right frontal cortex L, left/right parietal cortex L, and ACC. For both the SRTM2 and Logan reference model, regional coupling with the SRTM2 model was used to calculate the k_2_’ value. For the MRTM2 model, the MRTM model was used to calculate the k_2_’-value.

Finally, the possibility to perform a semi-quantitative compartmental analysis was investigated using the contrast of radioactivity (kBq/cc) in each ROI, over the radioactivity (kBq/cc) in the reference region, at different scan times (Eq.4):

4 $$ \mathrm{Activity}\ \mathrm{contrast}=\frac{\mathrm{Activity}\ \left(\mathrm{ROI}\right)}{\mathrm{Activity}\ \left(\mathrm{Cerbellum}\right)}-1 $$

This contrast was calculated by summing the time frames of four different 20-min intervals of the dynamic scan (10–30, 20–40, 30–50 and 40–60 min). Consequently, these values were plotted against the preferred compartmental model.

### Statistical analysis

Statistical analysis was similar to previously reported analysis by Pauwelyn et al. [34] using RStudio 1.1.456 with packages MASS (version 7.3–50) and Sommer (version 3.0). In summary: A multivariate linear mixed model with heterogeneous (unstructured) variances was set up on the data, containing BP_ND_ as response variable and the delineated ROI’s as outcome variable. Furthermore, the kinetic models were set as fixed factor and the individual animals as random factor. Using the Welsh-Satterthwaite equation, the degrees of freedom could be calculated and the type-I error α was set at 0.05 and a random intercept was included. Pearson correlation coefficients (R^2^) were calculated in Microsoft Office Excel (Microsoft office 365, version 2018).

## Supplementary information

**Additional file 1.** V_T_-values, AIC-values and BP_ND_-values. Regional V_T_-values, AIC-values and BP_ND_-values per compartmental model.

## Data Availability

The datasets used and/or analysed during the current study are available from the corresponding author on reasonable request.

## Abbreviations

1-TC: One-tissue compartmental model
2-TC: Two-tissue compartmental model
5-HT_2A_: serotonin_2A_ receptor
ACC: Anterior cingulate cortex
AIC: Akaike information criterion
BP_ND_: Non-displaceable binding potential
CAN: Acetonitrile
CT: Computed tomography
DMSO: Dimethyl sulfoxide
EOS: End of synthesis
HPLC: High pressure liquid chromatography
i.m.: Intramuscular
i.v.: intravenous
k_D_: affinity
MeOH: Methanol
MR: Magnetic resonance
MRTM: multilinear reference tissue model
MRTM2: 2-parameter multilinear reference tissue model
NaOAc: Sodium acetate
ND: Non-displaceable
PET: Positron emission tomography
ROI: Regions of interest
Rpm: Rotation per minute
RTM: Reference tissue model
SE: Standard errors
SRTM2: 2-step Simplified reference tissue model
TE: Echo time
THF: Tetrahydrofuran
TI: Inversion time
TR: Repetition time
t_R_: Retention time
V_ND_: Non-displaceable Volume of distribution
V_T_: Total Volume of distribution

## References

[1] Kroll Tina, Elmenhorst David, Matusch Andreas, Wedekind Franziska, Weisshaupt Angela, Beer Simone, Bauer Andreas (2013). Suitability of [18F]Altanserin and PET to Determine 5-HT2A Receptor Availability in the Rat Brain: In Vivo and In Vitro Validation of Invasive and Non-Invasive Kinetic Models. Molecular Imaging and Biology.

[2] Hoyer D, Hannon JP, Martin GR (2002). Molecular, pharmacological and functional diversity of 5-HT receptors. Pharmacol Biochem Behav.

[3] Riss PJ, Hong YT, Williamson D, Caprioli D, Sitnikov S, Ferrari V, et al. Validation and quantification of [18F] altanserin binding in the rat brain using blood input and reference tissue modeling. J Cereb Blood Flow Metab [Internet]. 2011;31(12):2334–42. Available from: http://www.pubmedcentral.nih.gov/articlerender.fcgi?artid=3323196&tool=pmcentrez&rendertype=abstract. [cited 2015 Sept 3]. 10.1038/jcbfm.2011.94 PMC3323196 21750562

[4] Elhwuegi AS (2004). Central monoamines and their role in major depression. Prog Neuro-Psychopharmacology Biol Psychiatry.

[5] Naughton M, Mulrooney JB, Leonard BE (2000). A review of the role of serotonin receptors in psychiatric disorders. Hum Psychopharmacol.

[6] Frokjaer VG, Mortensen EL, Nielsen FÅ, Haugbol S, Pinborg LH, Adams KH (2008). Frontolimbic serotonin 2A receptor binding in healthy subjects is associated with personality risk factors for affective disorder. Biol Psychiatry.

[7] Hurlemann R, Matusch A, Kuhn KU, Berning J, Elmenhorst D, Winz O (2008). 5-HT2A receptor density is decreased in the at-risk mental state. Psychopharmacology.

[8] Soloff PH, Price JC, Meltzer CC, Fabio A, Frank GK, Kaye WH (2007). 5HT2A receptor binding is increased in borderline personality disorder. Biol Psychiatry.

[9] Adams KH, Hansen ES, Pinborg LH, Hasselbach SG, Svarer C, Holm S (2005). Patients with obsessive-compulsive disorder have increased 5-HT 2A receptor binding in the caudate nuclei. Int J Neuropsychopharmacol.

[10] Chee IS, Lee SW, Kim JL, Wang SK, Shin YO, Shin SC (2001). 5-HT2A receptor gene promoter polymorphism –1438A/G and bipolar disorder. Psychiatr Genet.

[11] Bryson A, Carter O, Norman T, Kanaan R (2017). 5-HT2A agonists: a novel therapy for functional neurological disorders?. Int J Neuropsychopharmacol.

[12] Mintun MA, Sheline YI, Moerlein SM, Vlassenko AG, Huang Y, Snyder AZ (2004). Decreased hippocampal 5-HT2A receptor binding in major depressive disorder: in vivo measurement with [18F] altanserin positron emission tomography. Biol Psychiatry.

[13] Meltzer CC, Price JC, Mathis CA, Greer PJ, Cantwell MN, Houck PR (1999). PET imaging of serotonin type 2A receptors in late-life neuropsychiatric disorders. Am J Psychiatry.

[14] Kroll Tina, Elmenhorst David, Matusch Andreas, Celik A. Avdo, Wedekind Franziska, Weisshaupt Angela, Beer Simone, Bauer Andreas (2014). [18F]Altanserin and small animal PET: Impact of multidrug efflux transporters on ligand brain uptake and subsequent quantification of 5-HT2A receptor densities in the rat brain. Nuclear Medicine and Biology.

[15] L’Estrade ET, Hansen HD, Erlandsson M, Ohlsson TG, Knudsen GM, Herth MM (2018). Classics in neuroimaging: the serotonergic 2A receptor system - from discovery to modern molecular imaging. ACS Chem Neurosci.

[16] Herth MM, Knudsen GM (2015). Current radiosynthesis strategies for 5-HT<inf>2A</inf> receptor PET tracers. J Label Compd Radiopharm.

[17] Staley JK, Van Dyck CH, Tan P-Z, Al Tikriti M, Ramsby Q, Klump H (2001). Comparison of [^18^F] altanserin and [^18^F] deuteroaltanserin for PET imaging of serotonin<inf>2A</inf>receptors in baboon brain: pharmacological studies. Nucl Med Biol.

[18] Paterson LM, Kornum BR, Nutt DJ, Pike VW, Knudsen GM (2013). 5-HT Radioligands for human brain imaging with PET and SPECT. Med Res Rev.

[19] Lemaire C (1991). Cantineau, Robert Guillaume M, Plenevaux a, Christiaens L. fluorine- 18-Altanserin: a Radioligand for the study of serotonin receptors with PET: radiolabeling and in vivo biologic behavior in rats. J Nucl Med.

[20] Kristiansen H, Elfving B, Plenge P, Pinborg LH, Gillings N, Knudsen GM (2005). Binding characteristics of the 5-HT2A receptor antagonists altanserin and MDL 100907. Synapse..

[21] Tan P-Z, Baldwin R, Van Dyck C, Al-Tikriti M, Roth B, Khan N (1999). Characterization of radioactive metabolites of 5-HT2A receptor PET ligand [18F] altanserin in human and rodent. Nucl Med Biol.

[22] Hermanne JP, Franck G, Lemaire C, Degueldre C, Maquet P, Guillaume M (2011). Serotonin 5HT 2 receptor imaging in the human brain using positron emission tomography and a new Radioligand, [ 18 F]Altanserin: results in young Normal controls. J Cereb Blood Flow Metab.

[23] Pinborg Lars H, Adams Karen H, Svarer Claus, Holm Søren, Hasselbalch Steen G, Haugbøl Steven, Madsen Jacob, Knudsen Gitte M (2003). Quantification of 5-HT2A Receptors in the Human Brain Using [18F]Altanserin-PET and the Bolus/Infusion Approach. Journal of Cerebral Blood Flow & Metabolism.

[24] Price JC, Lopresti BJ, Meltzer CC, Smith GS, Mason NS, Huang Y (2001). Analyses of [18F] altanserin bolus injection PET data II: consideration of radiolabled metabolites in humans. Synapse..

[25] Overall KL (2000). Natural animal models of human psychiatric conditions: assessment of mechanisms and validity. Prog Neuro-Psychopharmacol Biol Psychiatry.

[26] Cyranoski D (2010). Pet Project. Nature..

[27] Peremans K, Audenaert K, Coopman F, Blanckaert P, Jacobs F, Otte A (2003). Estimates of regional cerebral blood flow and 5-HT2A receptor density in impulsive, aggressive dogs with99mTc-ECD and123I-5-I-R91150. Eur J Nucl Med Mol Imaging.

[28] Vermeire S, Audenaert K, Dobbeleir A, de Meester R, Vandermeulen E, Waelbers T (2009). Regional cerebral blood flow changes in dogs with anxiety disorders, measured with SPECT. Brain Imaging Behav.

[29] Vermeire S, Audenaert K, De Meester R, Vandermeulen E, Waelbers T, De Spiegeleer B (2012). Serotonin 2A receptor, serotonin transporter and dopamine transporter alterations in dogs with compulsive behaviour as a promising model for human obsessive-compulsive disorder. Psychiatry Res Neuroimaging.

[30] DeFelipe J (2011). The evolution of the brain, the human nature of cortical circuits, and intellectual creativity. Front Neuroanat.

[31] Vermeire Simon, Audenaert Kurt, De Meester Rudy, Vandermeulen Eva, Waelbers Tim, De Spiegeleer Bart, Eersels Jos, Dobbeleir André, Peremans Kathelijne (2011). Neuro-imaging the serotonin 2A receptor as a valid biomarker for canine behavioural disorders. Research in Veterinary Science.

[32] Waelbers T, Polis I, Vermeire S, Dobbeleir A, Eersels J, De Spiegeleer B (2013). 5-HT2A receptors in the feline brain: 123I-5-I-R91150 kinetics and the influence of ketamine measured with micro-SPECT. J Nucl Med.

[33] Price JC, Lopresti BJ, Mason NS, Holt DP, Huang Y, Mathis CA (2001). Analyses of [ 18 F ] Altanserin bolus injection PET data. I : Consideration of Radiolabeled Metabolites in Baboons. Synapse.

[34] Pauwelyn G, Vlerick L, Dockx R, Verhoeven J, Dobbeleir A, Peremans K, et al. PET quantification of [18F] MPPF in the canine brain using blood input and reference tissue modelling. Gelovani JG, editor. PLoS One [Internet]. 2019 Jun 11 [cited 2019 Aug 5];14(6):e0218237. Available from: http://dx.plos.org/10.1371/journal.pone.0218237 10.1371/journal.pone.0218237 PMC6559658 31185062

[35] Curry BB (2001). Animal models used in identifying gender-related differences. Int J Toxicol.

[36] Czerniak R (2001). Gender-based differences in pharmacokinetics in laboratory animal models. Int J Toxicol.

[37] Van Laeken N, Taylor O, Polis I, Neyt S, Kersemans K, Dobbeleir A (2016). In vivo evaluation of blood based and reference tissue based PET quantifications of [11C] DASB in the canine brain. PLoS One.

[38] DuSharma, S; Jacobs, HL; Sharma K. The canine brain in stereotaxic coordinates: full sections in frontal, sagittal and hirzontal planes. The MIT Press; 1970.

[39] Watabe H, Channing MA, Der MG, Adams R, Jagoda E, Herscovitch P, et al. ll Kinetic Analysis of the 5-HT2A Ligand [ C ] MDL 100 , 907. 2000;899–909. 10.1097/00004647-200006000-00002 10894173

[40] Schmidt KC, Turkheimer FE (2002). Kinetic modeling in positron emission tomography. Q J Nucl Med.

[41] Logan J, Fowler JS, Volkow ND, Wolf AP, Dewey SL, Schlyer DJ (1981). Graphical analysis of reversible Radioligand binding from time-activity measurements applied to [N-11C-methyl]-( − )-cocaine PET studies in human subjects. Blood..

[42] Innis RB, Cunningham VJ, Delforge J, Fujita M, Gjedde A, Gunn RN, et al. Consensus nomenclature for in vivo imaging of reversibly binding radioligands. J Cereb Blood Flow Metab [Internet]. 2007 Sep [cited 2014 Jan 21];27(9):1533–9. Available from: http://www.ncbi.nlm.nih.gov/pubmed/17519979. 10.1038/sj.jcbfm.9600493 17519979

[43] Akaike H (1974). A new look at the statistical Model identification. IEEE Trans Automat Contr.

[44] Wu Y, Carson RE (2002). Noise reduction in the simplified reference tissue model for neuroreceptor functional imaging. J Cereb Blood Flow Metab.

[45] Ichise M, Liow JS, Lu JQ, Takano A, Model K, Toyama H (2003). Linearized reference tissue parametric imaging methods: application to [11C] DASB positron emission tomography studies of the serotonin transporter in human brain. J Cereb Blood Flow Metab.

[46] Ziegler LD, Fan R, Desrosiers AE, Scherer NF. Distribution Volume Ratios Without Blood Sampling from Graphical Analysis of PET Data 1994;3(100):1823–39.

