# Supplementary material for: Kinetic analysis of [18F] altanserin bolus injection in the canine brain using PET imaging
Source: BMC Vet Res. 2019 Nov 21;15:415. doi: 10.1186/s12917-019-2165-5 (PMC6873736; doi:10.1186/s12917-019-2165-5)
Supplement: Supplementary file 1 — Additional file 1. VT-values, AIC-values and BPND-values. Regional VT-values, AIC-values and BPND-values per compartmental model. [file 12917_2019_2165_MOESM1_ESM.docx]

VT-, BP_ND_- and AIC-values (mean ± SD)(n=5) for the three modelling methods: 1-Tissue compartmental model (1-TC), 2-tissue compartmental model (2-TC), Logan plot,

|  | **1-TC** | | | **2-TC** | | | **Logan Plot** | | |  |
| --- | --- | --- | --- | --- | --- | --- | --- | --- | --- | --- |
| **Region** | **V_T_** | **AIC** | **BP_ND_** | **V_T_** | **AIC** | **BP_ND_** | | **V_T_** | **BP_ND_** | |
| Presubgenual cingulate gyrus | 10,0 ± 2.29 | 71 ± 14 | 1,72 ± 0.37 | 11.3 ± 2.69 | -11 ± 19 | 1,20 ± 0.19 | | 11,2 ± 2.76 | 1,11 ± 0.17 | |
| Subgenual cingulate gyrus | 10,1 ± 2.39 | 72 ± 15 | 1,72 ± 0.35 | 11.3 ± 2.86 | -3 ± 19 | 1,20 ± 0.18 | | 11,3 ± 2.87 | 1,12 ± 0.17 | |
| Frontal cortex L | 7,81 ± 1.65 | 73 ± 13 | 1,12 ± 0.22 | 8.89 ± 1.92 | -31 ± 37 | 0,74 ± 0.07 | | 8,88 ± 1.99 | 0,68 ± 0.08 | |
| Frontal cortex R | 7,68 ± 1.62 | 75 ± 15 | 1,08 ± 0.22 | 8.81 ± 1.93 | -18 ± 27 | 0,72 ± 0.08 | | 8,83 ± 1.93 | 0,67 ± 0.08 | |
| Temporal cortex L | 6,90 ± 1.48 | 72 ± 15 | 0,87 ± 0.20 | 7.99 ± 1.75 | -27 ± 36 | 0,56 ± 0.06 | | 7,93 ± 1.74 | 0,50 ± 0.06 | |
| Temporal cortex R | 6,73 ± 1.55 | 75 ± 12 | 0,82 ± 0.19 | 7.86 ± 1.89 | -22 ± 28 | 0,53 ± 0.08 | | 7,82 ± 1.84 | 0,47 ± 0.07 | |
| Occipital cortex L | 5,98 ± 1.40 | 83 ± 12 | 0,61 ± 0.16 | 7.35 ± 1.78 | -14 ± 29 | 0,43 ± 0.06 | | 7,35 ± 1.77 | 0,39 ± 0.06 | |
| Occipital cortex R | 5,90 ± 1.39 | 84 ± 11 | 0,59 ± 0.13 | 7.26 ± 1.83 | -17 ± 29 | 0,41 ± 0.05 | | 7,27 ± 1.79 | 0,37 ± 0.04 | |
| Parietal cortex L | 7,82 ± 1.87 | 70 ± 14 | 1,11 ± 0.26 | 8.98 ± 2.21 | -26 ± 33 | 0,75 ± 0.10 | | 8,84 ± 2.21 | 0,66 ± 0.08 | |
| Parietal cortex R | 7,85 ± 1.79 | 72 ± 13 | 1,12 ± 0.22 | 9.12 ± 2.38 | -28 ± 27 | 0,78 ± 0.09 | | 8,98 ± 2.18 | 0,69 ± 0.07 | |
| ACC | 9,29 ± 2.05 | 70 ± 17 | 1,52 ± 0.37 | 10.5 ± 2.38 | -18 ± 34 | 1,04 ± 0.18 | | 10,4 ± 2.47 | 0,96 ± 0.18 | |
| PCC | 6,90 ± 1.65 | 75 ± 18 | 0,87 ± 0.20 | 7.93 ± 2.04 | 0 ± 21 | 0,54 ± 0.10 | | 8,01 ± 2.10 | 0,51 ± 0.11 | |
| Cerebellum | 3,71 ± 0.81 | 89 ± 12 | / | 5.12 ± 1.14 | 13 ± 19 | / | | 5,31 ± 1.25 | / | |

BP_ND_ values (mean ± SD)(n=5) for the reference tissue models (RTM): Simplified reference tissue model 2 (SRTM2), Logan reference tissue model and Multilinear reference tissue model 2 (MRTM2).

|  | **SRTM2** | **Logan reference** | **MRTM2** |
| --- | --- | --- | --- |
| **Region** | **BP_ND_** | **BP_ND_** | **BP_ND_** |
| Presubgenual cingulate gyrus | 1,11 ± 0.19 | 1,13 ± 0.19 | 1,12 ± 0.20 |
| Subgenual cingulate gyrus | 1,14 ± 0.18 | 1,15 ± 0.19 | 1,15 ± 0.19 |
| Frontal cortex L | 0,69 ± 0.09 | 0,71 ± 0.09 | 0,69 ± 0.09 |
| Frontal cortex R | 0,66 ± 0.07 | 0,69 ± 0.07 | 0,67 ± 0.07 |
| Temporal cortex L | 0,54 ± 0.09 | 0,53 ± 0.07 | 0,52 ± 0.06 |
| Temporal cortex R | 0,50 ± 0.06 | 0,51 ± 0.06 | 0,50 ± 0.07 |
| Occipital cortex L | 0,39 ± 0.07 | 0,40 ± 0.07 | 0,40 ± 0.07 |
| Occipital cortex R | 0,37 ± 0.05 | 0,38 ± 0.05 | 0,39 ± 0.07 |
| Parietal cortex L | 0,74 ± 0.12 | 0,71 ± 0.09 | 0,71 ± 0.10 |
| Parietal cortex R | 0,75 ± 0.08 | 0,74 ± 0.08 | 0,74 ± 0.08 |
| ACC | 0,95 ± 0.18 | 0,98 ± 0.18 | 0,96 ± 0.19 |
| PCC | 0,49 ± 0.11 | 0,51 ± 0.11 | 0,50 ± 0.11 |
